# (6-Acetyl-1,3,7-trimethyl­lumazine-κ^3^ *O* ^4^,*N* ^5^,*O* ^6^)bis­(triphenyl­phosphine-κ*P*)copper(I) hexa­fluorido­phosphate

**DOI:** 10.1107/S1600536810000735

**Published:** 2010-01-13

**Authors:** Francisco Hueso-Ureña, Nuria A. Illán-Cabeza, Sonia B. Jiménez-Pulido, Miguel N. Moreno-Carretero

**Affiliations:** a Departamento de Química Inorgánica y Orgánica, Facultad de Ciencias Experimentales, Campus Universitario Las Lagunillas (B3), Universidad de Jaén, 23071 Jaén, Spain

## Abstract

The title compound, [Cu(C_11_H_12_N_4_O_3_)(C_18_H_15_P)_2_]PF_6_, is the third example reported in the literature of a five-coordinated Cu^I^P_2_NO_2_ system. The metal is coordinated to both PPh_3_ mol­ecules through the P atoms and to the pyrazine ring of the lumazine mol­ecule through an N atom in a trigonal–planar arrangement; two additional coordinated O atoms, at Cu—O distances longer than 2.46 Å, complete the coordination. The coordination environment can be described as an inter­mediate square-pyramidal/trigonal–bipyramidal (SP/TBP) polyhedron.

## Related literature

For related literature on the coordination behaviour of pteridine and related ligands, see: Jiménez Pulido *et al.* (2001 ▶, 2008 ▶); Acuña-Cueva *et al.* (2003 ▶); Hueso-Ureña *et al.* (2008 ▶). For related literature on similar Cu^I^ coordination environments, see: Wanner *et al.* (1999 ▶); Hueso-Ureña *et al.* (2008 ▶). For additional structural details quoted in the comment, see: Addison *et al.* (1984 ▶); Cremer & Pople (1975 ▶); Janiak (2000 ▶); Muetterties & Guggenberger (1974 ▶); Spek (2009 ▶).
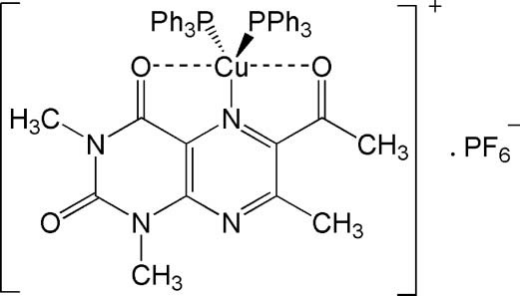


## Experimental

### 

#### Crystal data

[Cu(C_11_H_12_N_4_O_3_)(C_18_H_15_P)_2_]PF_6_
*M* *_r_* = 981.3
Monoclinic, 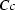

*a* = 10.2627 (13) Å
*b* = 26.890 (3) Å
*c* = 15.5387 (15) Å
β = 92.666 (10)°
*V* = 4283.5 (8) Å^3^
*Z* = 4
Mo *K*α radiation
μ = 0.70 mm^−1^
*T* = 293 K
0.26 × 0.14 × 0.14 mm

#### Data collection

Nonius KappaCCD diffractometer
Absorption correction: multi-scan (*SADABS*; Sheldrick, 2003 ▶) *T*_min_ = 0.840, *T*_max_ = 0.909
32442 measured reflections
9626 independent reflections
6616 reflections with *I* > 2σ(*I*)
*R*_int_ = 0.066

#### Refinement

*R*[*F* ^2^ > 2σ(*F* ^2^)] = 0.049
*wR*(*F* ^2^) = 0.113
*S* = 1.04
9626 reflections
581 parameters
2 restraints
H-atom parameters constrained
Δρ_max_ = 0.44 e Å^−3^
Δρ_min_ = −0.45 e Å^−3^
Absolute structure: Flack (1983 ▶)
Flack parameter: 0.05 (2)

### 

Data collection: *COLLECT* (Nonius, 1998 ▶); cell refinement: *DIRAX/LSQ* (Duisenberg, 1992 ▶); data reduction: *EVALCCD* (Duisenberg *et al.*, 2003 ▶); program(s) used to solve structure: *SIR92* (Altomare *et al.*, 1994 ▶); program(s) used to refine structure: *SHELXL97* (Sheldrick, 2008 ▶); molecular graphics: *Mercury* (Macrae *et al.*, 2006 ▶); software used to prepare material for publication: *WinGX* (Farrugia, 1999 ▶).

## Supplementary Material

Crystal structure: contains datablocks I, global. DOI: 10.1107/S1600536810000735/bg2316sup1.cif

Structure factors: contains datablocks I. DOI: 10.1107/S1600536810000735/bg2316Isup2.hkl

Additional supplementary materials:  crystallographic information; 3D view; checkCIF report

## Figures and Tables

**Table 1:** Selected bond lengths (Å)

Cu—N5	2.036 (3)
Cu—P2	2.212 (1)
Cu—P1	2.238 (1)
Cu—O4	2.466 (3)
Cu—O61	2.529 (3)

**Fig. 1.**
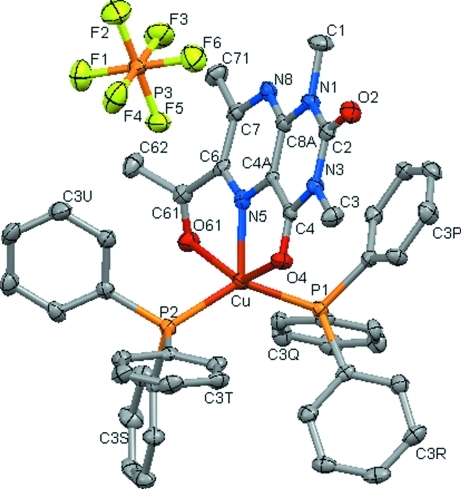
View of the molecular unit of [Cu(DLMAceM)(PPh3)2]PF6 (ellipsoids at 50% probability). Atoms from the pteridine moiety have been labelled following IUPAC numbering system. For clarity, H atoms have been ommited. In each phenyl ring, only the C3 atom has been labelled, the C1 being bound to phosphorus.

## supplementary crystallographic information

### Comment

Five-coordinated copper(I) complexes are very rare as contrasted with the copper(II) ones. A bibliographical survey using the CCDC database indicates only two previously reported Cu^I^P_2_NO_2_ examples: the Cu(I) complex with the 2',7',9'-trimethylesther of pyrroloquinoline-quinone (Wanner *et al.*, 1999) and the isostructural perchlorate salt of the title compound (Hueso-Ureña *et al.*, 2008). The title compound shows a salt-like structure containing [Cu(DLMAceM)(PPh_3_)_2_]^+^ cations (DLMAceM = 6-acetyl-1,3,7-trimethylpteridine-2,4(1*H*,3*H*)-dione, see Scheme 1) and octahedral hexafluorophosphate anions (Fig. 1).

Within the relative arrangement of the acetyl group and the pteridine moiety (N5—C6—C61—O61 torsion: 166.5 (2)°), the free DLMAceM (Hueso-Ureña *et al.*, 2008) is not able to act as tridentate ligand through the O4, N5 and O61 atoms; however, the energy difference between the metal-free conformation and the Cu(I)-coordinated one (N5—C6—C61—O61 torsion, 31.9 (7)°), in which the acetyl mean plane is turned off by *ca*133° around the C6—C61 bond, is not large enough to avoid the formation of M—*L* bonds, despite the steric hindrances between C62 and C71 methyl groups. Due to the coordination steric requirements, both rings of the pteridine moiety are slightly angled (7.4 (2)°). The two five-membered chelates are also angled to each other by 10.3 (2)°. Despite the fact that the Cremer and Pople's ring puckering analysis (Cremer & Pople, 1975) may be dubious since the bond distance range/average is higher than 25%, the closest description of the chelate-rings could be as a half-chair twisted on N5—Cu (Cu—N5—C4A—C4—O4, *Φ*=169.3° and *k*=4.20) and an envelope on C61 (Cu—N5—C6—C61—O61, *Φ*=253.9° and *k*=7.05). The metal is trigonal-planar three-coordinated with atoms P1, P2 and N5 (Cu—P1, 2.238 (1); Cu—P2, 2.212 (1); Cu—N5, 2.036 (3) Å). The metal center lies only slightly out of the plane defined by the three donor atoms (0.021 Å); despite bond angles around the metal (N5—Cu—P1, 108.2 (1); N5—Cu—P2, 121.1 (1); P1—Cu—P2, 130.68 (4)°) deviate by a little extent from the ideal value (120°), their sum is 360.0°.

The lumazine ligand is arranged in a roughly perpendicular fashion (81.0°) to the above-mentioned trigonal plane, the metal ion lying 0.41 Å out of this plane; in this plane, there are two additional and very long Cu···O4 and Cu···O61 (2.466 (3) and 2.529 (3) Å, respectively). The final coordination polyhedron can be defined as an intermediate TBP/SP shaped polyhedron, since Addison's τ criterion (Addison *et al.*, 1984) indicates a distorted square-pyramid (τ= 1/5, apical atom N5), whereas Muetterties and Guggenberger's calculation (Muetterties & Guggenberger, 1974) shows a distorted trigonal-bipyramid (Δ = 1/5) with an P1/P2/N5 equatorial plane and a skewed O61···Cu···O4 axis due the restricted *bite* of tridentate DLMAceM. This description is coincident with those previously reported for the related perchlorate compound (Hueso-Ureña *et al.*, 2008).

A close comparison of the coordination environments of the title compound and its related perchlorate one (Hueso-Ureña *et al.*, 2008) with the Cu(I) complex of the 2',7',9'-trimethylesther of pyrroloquinoline-quinone (Wanner *et al.*, 1999) indicates that both DLMAceM Cu(I) complexes show a little more unsymmetrical PPh_3_ groups with a P1 atom occupying a somewhat more apical position than P2; thus, the difference between both Cu—P bond lengths in the DLMAceM complexes (0.034 Å for Cu/DLMAceM/ClO_4_ and 0.026 Å for the title compound) is higher than in those reported by Kaim (0.014 Å) (Wanner *et al.*, 1999). In addition to this, whereas the exocyclic carbonyl Cu···O distances are similar (2.559 (5) and 2.529 (3) Å for Cu/DLMAceM/ClO_4_ and the title compound and 2.579 (4)Å for the Wanner's compound), the endocyclic ones are quiet different (2.479 (5), 2.466 (3) Å and 2.254 (4) Å, respectively), the very weak O4-DLMAceM semicoordinative behaviour being in according with previously reported results for analogous lumazine derivatives (Acuña-Cueva *et al.*, 2003; Jiménez-Pulido *et al.*, 2008).

The analysis of short π-π ring interactions, made with *PLATON* (Spek, 2009), in the crystal structure does not indicate the existence of any significative π-stacking interaction. Only the interaction between both pyrimidine and pyrazine rings from DLMAceM and the P phenyl ring from PPh_3_ could be cited, but despite distances between centroids (3.696 (3) and 4.039 (3) Å) lies in the range accepted for these interactions (3.4–4.6 Å), the interplanar dihedral angles α (15.6 and 14.7°, respectively) clearly show both rings of each couple are far to be parallel enough to consider the existence of π-stacking (Janiak, 2000).

The packing structure indicates that hexafluorophosphate anions are placed in cavities and held in place by a large number of F···H—C interactions at distances smaller than the sum of the Van der Waals' radii (2.67 Å), F···H lengths ranging from 2.46 to 2.55 Å and F···H—C angles, from 118 to 144°; the arrangement of the counteranions is similar than in the crystal structure of the related perchlorate compound (Hueso-Ureña *et al.*, 2008), but in the latter one there are fewer interactions, which justifies the disorder of these anions. Finally, in both structures, unit cell contains no additional residual solvent accessible voids.

The structure of the Cu(I) complex cation in this hexafluorophosphate salt is similar to the previously reported for the perchlorate analog (Hueso-Ureña *et al.*, 2008), in which the nature of the metal-ligand bonds, especially in regard to the semicoordinated oxygen atoms, was defined using an AIM topological analysis of the electron density. The results indicated that Cu—*X* (*X* = N, O, P) bonds are weakly closed-shell interactions (Hueso-Ureña *et al.*, 2008). On the other hand, the delocalization indices for the Cu···O bonds are lower than the Cu—P values, which is related to the higher covalent character of the Cu—P bonds *versus* the Cu···O interactions, the Cu—N interaction being intermediate between both.

### Experimental

Single-crystals of the title compound were easily obtained by adding sodium hexafluorophosphate (2 mmol) to an aqueous solution (40 ml) of the isomorphic bis-triphenylphosphine-(*N*^5^,*O*^4^,*O*^61^)-6-acetyl-1,3,7-trimethyl-pteridine-2,4(1*H*,3*H*)-dione-copper(I) perchlorate (1 mmol), previously synthesized and characterized (Hueso-Ureña *et al.*, 2008).

### Refinement

All H atoms were treated as riding, with C—H (methyl) = 0.96 Å (*U*ĩso~(H) = 1.5*U*~eq~(C)) and C—H (aromatic) = 0.93 Å (*U*ĩso~(H) = 1.2*U*~eq~(C)).

### Crystal data

**Table:**

[Cu(C_11_H_12_N_4_O_3_)(C_18_H_15_P)_2_]PF_6_	*F*(000) = 2016
*M**_r_* = 981.3	*D*_x_ = 1.522 Mg m^−^^3^
Monoclinic, *C**c*	Mo *K*α radiation, λ = 0.71073 Å
Hall symbol: C -2yc	Cell parameters from 54 reflections
*a* = 10.2627 (13) Å	θ = 1.5–27.5°
*b* = 26.890 (3) Å	µ = 0.70 mm^−^^1^
*c* = 15.5387 (15) Å	*T* = 293 K
β = 92.666 (10)°	Prism, red
*V* = 4283.5 (8) Å^3^	0.26 × 0.14 × 0.14 mm
*Z* = 4	

### Data collection

**Table:**

Nonius KappaCCD diffractometer	9626 independent reflections
Radiation source: Enraf–Nonius FR590	6616 reflections with *I* > 2σ(*I*)
graphite	*R*_int_ = 0.066
Detector resolution: 9 pixels mm^-1^	θ_max_ = 27.5°, θ_min_ = 1.5°
CCD rotation images, thick slices scans	*h* = −13→13
Absorption correction: multi-scan (*SADABS*; Sheldrick, 2003)	*k* = −34→34
*T*_min_ = 0.840, *T*_max_ = 0.909	*l* = −20→20
32442 measured reflections	

### Refinement

**Table:**

Refinement on *F*^2^	Secondary atom site location: difference Fourier map
Least-squares matrix: full	Hydrogen site location: inferred from neighbouring sites
*R*[*F*^2^ > 2σ(*F*^2^)] = 0.049	H-atom parameters constrained
*wR*(*F*^2^) = 0.113	*w* = 1/[σ^2^(*F*_o_^2^) + (0.0499*P*)^2^] where *P* = (*F*_o_^2^ + 2*F*_c_^2^)/3
*S* = 1.04	(Δ/σ)_max_ < 0.001
9626 reflections	Δρ_max_ = 0.44 e Å^−^^3^
581 parameters	Δρ_min_ = −0.45 e Å^−^^3^
2 restraints	Absolute structure: Flack (1983)
Primary atom site location: structure-invariant direct methods	Flack parameter: 0.05 (2)

### Special details

**Table:**

Geometry. All e.s.d.'s (except the e.s.d. in the dihedral angle between two l.s. planes) are estimated using the full covariance matrix. The cell e.s.d.'s are taken into account individually in the estimation of e.s.d.'s in distances, angles and torsion angles; correlations between e.s.d.'s in cell parameters are only used when they are defined by crystal symmetry. An approximate (isotropic) treatment of cell e.s.d.'s is used for estimating e.s.d.'s involving l.s. planes.
Refinement. Refinement of *F*^2^ against ALL reflections. The weighted *R*-factor *wR* and goodness of fit *S* are based on *F*^2^, conventional *R*-factors *R* are based on *F*, with *F* set to zero for negative *F*^2^. The threshold expression of *F*^2^ > σ(*F*^2^) is used only for calculating *R*-factors(gt) *etc*. and is not relevant to the choice of reflections for refinement. *R*-factors based on *F*^2^ are statistically about twice as large as those based on *F*, and *R*- factors based on ALL data will be even larger.

### Fractional atomic coordinates and isotropic or equivalent isotropic displacement parameters (Å^2^)

**Table:**

	*x*	*y*	*z*	*U*_iso_*/*U*_eq_	
Cu	0.22607 (4)	0.126244 (17)	0.60587 (3)	0.02174 (14)	
N1	0.2716 (3)	−0.06843 (13)	0.5940 (2)	0.0202 (8)	
C1	0.3402 (5)	−0.11563 (17)	0.5769 (3)	0.0339 (13)	
C2	0.1484 (4)	−0.07141 (17)	0.6241 (3)	0.0238 (10)	
O2	0.0929 (3)	−0.11007 (12)	0.6336 (2)	0.0290 (8)	
N3	0.0876 (3)	−0.02665 (13)	0.6427 (2)	0.0207 (8)	
C3	−0.0435 (4)	−0.03073 (18)	0.6760 (3)	0.0285 (11)	
C4	0.1403 (4)	0.01970 (16)	0.6319 (3)	0.0199 (10)	
O4	0.0815 (3)	0.05801 (11)	0.6478 (2)	0.0250 (7)	
C4A	0.2728 (4)	0.01959 (16)	0.6034 (3)	0.0170 (9)	
N5	0.3348 (3)	0.06312 (13)	0.6001 (2)	0.0203 (8)	
C6	0.4595 (4)	0.06272 (16)	0.5819 (3)	0.0193 (9)	
C61	0.5246 (4)	0.11170 (17)	0.5938 (3)	0.0230 (10)	
O61	0.4605 (3)	0.14889 (11)	0.5807 (2)	0.0284 (7)	
C62	0.6600 (4)	0.11408 (18)	0.6316 (3)	0.0355 (12)	
C7	0.5198 (4)	0.01815 (17)	0.5577 (3)	0.0230 (10)	
C71	0.6574 (4)	0.01566 (18)	0.5317 (3)	0.0330 (12)	
N8	0.4553 (4)	−0.02503 (14)	0.5563 (2)	0.0238 (9)	
C8A	0.3351 (4)	−0.02434 (16)	0.5839 (3)	0.0202 (10)	
P1	0.11399 (10)	0.13528 (4)	0.47983 (7)	0.0193 (2)	
C1P	0.0969 (4)	0.07510 (15)	0.4299 (3)	0.0192 (9)	
C2P	−0.0028 (4)	0.04297 (16)	0.4482 (3)	0.0256 (11)	
C3P	−0.0016 (5)	−0.00556 (17)	0.4194 (3)	0.0316 (12)	
C4P	0.0995 (5)	−0.02251 (17)	0.3736 (3)	0.0298 (11)	
C5P	0.2003 (4)	0.00815 (17)	0.3557 (3)	0.0271 (11)	
C6P	0.1985 (4)	0.05694 (16)	0.3826 (3)	0.0243 (10)	
C1Q	0.1852 (4)	0.17328 (15)	0.3980 (3)	0.0205 (10)	
C2Q	0.2899 (4)	0.20335 (16)	0.4201 (3)	0.0237 (10)	
C3Q	0.3385 (4)	0.23579 (18)	0.3614 (3)	0.0300 (11)	
C4Q	0.2840 (4)	0.23803 (17)	0.2787 (3)	0.0289 (11)	
C5Q	0.1812 (4)	0.20761 (17)	0.2558 (3)	0.0274 (11)	
C6Q	0.1318 (4)	0.17536 (16)	0.3149 (3)	0.0231 (10)	
C1R	−0.0479 (4)	0.16222 (16)	0.4829 (3)	0.0191 (9)	
C2R	−0.1535 (4)	0.14940 (18)	0.4283 (3)	0.0264 (11)	
C3R	−0.2694 (4)	0.17489 (18)	0.4320 (3)	0.0315 (12)	
C4R	−0.2812 (5)	0.21349 (17)	0.4890 (3)	0.0326 (12)	
C5R	−0.1797 (4)	0.22681 (17)	0.5421 (3)	0.0278 (11)	
C6R	−0.0628 (4)	0.20129 (16)	0.5402 (3)	0.0256 (10)	
P2	0.23582 (10)	0.17405 (4)	0.72188 (7)	0.0191 (2)	
C1S	0.2300 (4)	0.24038 (15)	0.6999 (3)	0.0205 (9)	
C2S	0.3286 (5)	0.26168 (17)	0.6541 (3)	0.0287 (11)	
C3S	0.3284 (5)	0.31132 (18)	0.6367 (3)	0.0357 (12)	
C4S	0.2258 (5)	0.34057 (17)	0.6621 (3)	0.0365 (12)	
C5S	0.1278 (5)	0.31999 (17)	0.7051 (3)	0.0317 (12)	
C6S	0.1294 (4)	0.27024 (17)	0.7250 (3)	0.0267 (11)	
C1T	0.1076 (4)	0.16277 (15)	0.7964 (3)	0.0195 (9)	
C2T	−0.0107 (4)	0.14380 (16)	0.7660 (3)	0.0228 (10)	
C3T	−0.1053 (4)	0.13098 (17)	0.8232 (3)	0.0260 (10)	
C4T	−0.0799 (4)	0.13703 (17)	0.9103 (3)	0.0291 (11)	
C5T	0.0370 (4)	0.15600 (17)	0.9411 (3)	0.0278 (11)	
C6T	0.1297 (4)	0.16895 (15)	0.8839 (3)	0.0228 (10)	
C1U	0.3795 (4)	0.16716 (16)	0.7928 (3)	0.0207 (10)	
C2U	0.4230 (4)	0.11905 (17)	0.8091 (3)	0.0252 (10)	
C3U	0.5174 (5)	0.10979 (19)	0.8722 (3)	0.0338 (12)	
C4U	0.5729 (4)	0.1486 (2)	0.9180 (3)	0.0333 (12)	
C5U	0.5342 (4)	0.19633 (19)	0.9008 (3)	0.0318 (12)	
C6U	0.4372 (4)	0.20573 (17)	0.8385 (3)	0.0260 (11)	
P3	0.58388 (12)	−0.03276 (5)	0.78832 (8)	0.0276 (3)	
F1	0.6892 (3)	0.00507 (12)	0.82619 (19)	0.0503 (8)	
F2	0.6615 (3)	−0.07689 (12)	0.8341 (2)	0.0613 (10)	
F3	0.6624 (3)	−0.04046 (13)	0.70555 (19)	0.0535 (9)	
F4	0.5035 (3)	−0.02591 (12)	0.87252 (18)	0.0495 (8)	
F5	0.5034 (3)	0.01136 (10)	0.74542 (18)	0.0427 (8)	
F6	0.4772 (3)	−0.07066 (11)	0.7511 (2)	0.0519 (8)	
H1A	0.3312	−0.1233	0.5166	0.051*	
H1B	0.3029	−0.142	0.6094	0.051*	
H1C	0.431	−0.1122	0.5937	0.051*	
H3A	−0.0755	0.0018	0.689	0.043*	
H3B	−0.0399	−0.0506	0.7274	0.043*	
H3C	−0.101	−0.0461	0.6334	0.043*	
H62A	0.7204	0.1136	0.5863	0.053*	
H62B	0.6759	0.086	0.6687	0.053*	
H62C	0.6713	0.1442	0.6644	0.053*	
H71A	0.715	0.0161	0.5822	0.05*	
H71B	0.6756	0.0438	0.4961	0.05*	
H71C	0.6704	−0.0144	0.5	0.05*	
H2P	−0.0713	0.0541	0.4802	0.031*	
H3P	−0.0701	−0.0268	0.4312	0.038*	
H4P	0.0997	−0.0553	0.3545	0.036*	
H5P	0.2699	−0.0038	0.3254	0.033*	
H6P	0.2662	0.0781	0.3689	0.029*	
H2Q	0.3282	0.2017	0.4755	0.028*	
H3Q	0.4085	0.2563	0.3775	0.036*	
H4Q	0.3167	0.26	0.2389	0.035*	
H5Q	0.1446	0.2087	0.1999	0.033*	
H6Q	0.0619	0.1548	0.2987	0.028*	
H2R	−0.1459	0.1235	0.3891	0.032*	
H3R	−0.3402	0.1659	0.3958	0.038*	
H4R	−0.3598	0.2306	0.491	0.039*	
H5R	−0.1882	0.2532	0.5801	0.033*	
H6R	0.0065	0.2104	0.5776	0.031*	
H2S	0.3956	0.2418	0.6351	0.034*	
H3S	0.3967	0.3255	0.608	0.043*	
H4S	0.2244	0.3744	0.6496	0.044*	
H5S	0.0587	0.3397	0.7214	0.038*	
H6S	0.0622	0.2566	0.7556	0.032*	
H2T	−0.0272	0.1396	0.7071	0.027*	
H3T	−0.1852	0.1184	0.8027	0.031*	
H4T	−0.1428	0.1281	0.9487	0.035*	
H5T	0.0535	0.1601	1	0.033*	
H6T	0.2088	0.1821	0.9047	0.027*	
H2U	0.3876	0.0928	0.7768	0.03*	
H3U	0.5438	0.0773	0.884	0.041*	
H4U	0.6371	0.1423	0.9609	0.04*	
H5U	0.5734	0.2226	0.9312	0.038*	
H6U	0.4108	0.2383	0.8273	0.031*	

### Atomic displacement parameters (Å^2^)

**Table:**

	*U*^11^	*U*^22^	*U*^33^	*U*^12^	*U*^13^	*U*^23^
Cu	0.0251 (3)	0.0193 (3)	0.0208 (3)	0.0017 (3)	0.0003 (2)	−0.0014 (3)
P1	0.0198 (6)	0.0182 (6)	0.0201 (6)	−0.0016 (5)	0.0019 (5)	−0.0011 (5)
P3	0.0303 (7)	0.0282 (7)	0.0240 (7)	0.0092 (6)	−0.0019 (5)	0.0004 (6)
P2	0.0207 (6)	0.0170 (6)	0.0197 (6)	−0.0007 (5)	0.0016 (5)	−0.0002 (5)
F5	0.0552 (18)	0.0388 (18)	0.0334 (17)	0.0198 (15)	−0.0044 (14)	−0.0035 (14)
F4	0.0354 (17)	0.085 (2)	0.0288 (16)	0.0019 (16)	0.0031 (13)	0.0025 (16)
O2	0.0344 (19)	0.0212 (17)	0.0312 (19)	−0.0059 (16)	−0.0004 (15)	0.0040 (15)
F3	0.0449 (18)	0.077 (2)	0.0400 (19)	0.0188 (17)	0.0119 (15)	−0.0074 (17)
F1	0.0419 (18)	0.058 (2)	0.050 (2)	−0.0132 (16)	−0.0049 (14)	−0.0048 (17)
O4	0.0258 (17)	0.0189 (17)	0.0309 (19)	0.0019 (14)	0.0061 (14)	0.0000 (14)
F2	0.074 (2)	0.047 (2)	0.061 (2)	0.0180 (18)	−0.0148 (19)	0.0068 (17)
O61	0.0243 (17)	0.0264 (18)	0.035 (2)	−0.0006 (15)	0.0027 (14)	0.0011 (15)
F6	0.0561 (19)	0.0426 (19)	0.056 (2)	−0.0042 (15)	−0.0058 (17)	−0.0024 (15)
N3	0.021 (2)	0.018 (2)	0.023 (2)	−0.0033 (16)	−0.0001 (16)	0.0019 (16)
N1	0.0220 (19)	0.0152 (19)	0.024 (2)	−0.0008 (15)	0.0026 (16)	−0.0012 (16)
N8	0.027 (2)	0.027 (2)	0.018 (2)	−0.0007 (18)	0.0017 (16)	−0.0039 (17)
N5	0.0235 (19)	0.021 (2)	0.0162 (19)	−0.0047 (17)	0.0012 (16)	0.0013 (16)
C8A	0.023 (2)	0.023 (3)	0.015 (2)	0.000 (2)	−0.0001 (18)	−0.0029 (19)
C6U	0.022 (2)	0.025 (3)	0.031 (3)	−0.006 (2)	0.006 (2)	−0.007 (2)
C2U	0.025 (2)	0.028 (3)	0.023 (2)	0.002 (2)	0.002 (2)	−0.002 (2)
C7	0.022 (2)	0.033 (3)	0.014 (2)	0.005 (2)	0.0009 (18)	0.004 (2)
C1Q	0.018 (2)	0.021 (2)	0.023 (2)	0.0025 (19)	0.0026 (18)	0.0007 (19)
C1U	0.019 (2)	0.024 (2)	0.020 (2)	−0.0006 (19)	0.0050 (18)	−0.002 (2)
C1R	0.018 (2)	0.022 (2)	0.018 (2)	0.0008 (19)	0.0022 (18)	0.0086 (19)
C1P	0.018 (2)	0.022 (2)	0.018 (2)	0.0010 (19)	−0.0003 (18)	0.0009 (19)
C5S	0.043 (3)	0.027 (3)	0.025 (3)	0.009 (2)	0.002 (2)	0.002 (2)
C2T	0.022 (2)	0.020 (2)	0.026 (3)	0.0031 (19)	0.001 (2)	0.0044 (19)
C4A	0.017 (2)	0.020 (2)	0.013 (2)	0.0008 (18)	−0.0021 (18)	0.0005 (18)
C4	0.025 (2)	0.021 (2)	0.013 (2)	−0.001 (2)	−0.0026 (18)	−0.0015 (19)
C71	0.022 (2)	0.041 (3)	0.036 (3)	0.003 (2)	0.003 (2)	0.001 (2)
C6Q	0.026 (2)	0.021 (2)	0.022 (3)	−0.0061 (19)	0.0000 (19)	−0.0009 (19)
C2Q	0.021 (2)	0.025 (2)	0.025 (2)	−0.001 (2)	−0.0004 (19)	−0.004 (2)
C6R	0.030 (3)	0.023 (2)	0.024 (3)	0.003 (2)	0.000 (2)	0.004 (2)
C2	0.031 (3)	0.024 (3)	0.017 (2)	0.002 (2)	−0.003 (2)	0.002 (2)
C3Q	0.030 (3)	0.029 (3)	0.031 (3)	−0.011 (2)	0.005 (2)	0.001 (2)
C1T	0.021 (2)	0.014 (2)	0.024 (2)	0.0045 (18)	0.0005 (18)	0.0039 (19)
C5Q	0.034 (3)	0.028 (3)	0.020 (2)	−0.003 (2)	0.003 (2)	0.002 (2)
C6S	0.028 (2)	0.022 (3)	0.029 (3)	0.001 (2)	0.003 (2)	0.003 (2)
C5R	0.034 (3)	0.024 (2)	0.026 (3)	0.007 (2)	0.010 (2)	0.004 (2)
C4T	0.023 (2)	0.035 (3)	0.030 (3)	0.006 (2)	0.010 (2)	0.007 (2)
C2S	0.033 (3)	0.026 (3)	0.028 (3)	0.000 (2)	0.008 (2)	0.005 (2)
C5T	0.027 (3)	0.036 (3)	0.020 (2)	0.008 (2)	0.004 (2)	0.003 (2)
C6	0.017 (2)	0.026 (2)	0.015 (2)	0.0037 (19)	−0.0016 (17)	0.0050 (19)
C1S	0.022 (2)	0.019 (2)	0.020 (2)	−0.0030 (19)	−0.0022 (18)	−0.0006 (18)
C6T	0.023 (2)	0.021 (2)	0.025 (2)	0.0044 (19)	−0.0020 (19)	0.001 (2)
C62	0.028 (3)	0.032 (3)	0.046 (3)	−0.008 (2)	−0.008 (2)	0.008 (2)
C1	0.036 (3)	0.018 (3)	0.048 (3)	0.006 (2)	0.005 (2)	−0.002 (2)
C3P	0.027 (3)	0.030 (3)	0.038 (3)	−0.008 (2)	0.004 (2)	−0.003 (2)
C4Q	0.029 (3)	0.025 (3)	0.034 (3)	−0.004 (2)	0.010 (2)	0.005 (2)
C2P	0.024 (2)	0.024 (3)	0.029 (3)	0.002 (2)	0.007 (2)	−0.002 (2)
C4R	0.034 (3)	0.028 (3)	0.037 (3)	0.012 (2)	0.009 (2)	0.012 (2)
C5U	0.021 (2)	0.039 (3)	0.035 (3)	−0.007 (2)	−0.001 (2)	−0.005 (2)
C4P	0.038 (3)	0.019 (2)	0.032 (3)	0.000 (2)	0.001 (2)	−0.006 (2)
C3T	0.019 (2)	0.026 (3)	0.034 (3)	0.0033 (19)	0.0042 (19)	0.000 (2)
C3	0.020 (2)	0.028 (3)	0.038 (3)	−0.002 (2)	0.004 (2)	0.004 (2)
C3S	0.042 (3)	0.024 (3)	0.041 (3)	−0.007 (2)	0.006 (2)	0.004 (2)
C6P	0.023 (2)	0.022 (2)	0.027 (3)	−0.002 (2)	0.000 (2)	0.001 (2)
C4U	0.019 (2)	0.050 (3)	0.030 (3)	0.005 (2)	−0.003 (2)	−0.001 (2)
C4S	0.060 (3)	0.016 (3)	0.033 (3)	−0.004 (3)	−0.005 (3)	0.001 (2)
C3U	0.036 (3)	0.035 (3)	0.030 (3)	0.014 (2)	0.000 (2)	0.002 (2)
C5P	0.026 (3)	0.025 (3)	0.030 (3)	0.002 (2)	−0.001 (2)	−0.007 (2)
C3R	0.020 (2)	0.038 (3)	0.036 (3)	0.003 (2)	0.001 (2)	0.009 (2)
C2R	0.027 (3)	0.028 (3)	0.024 (3)	0.001 (2)	0.001 (2)	0.000 (2)
C61	0.023 (2)	0.027 (3)	0.019 (2)	0.000 (2)	0.0045 (19)	0.003 (2)

### Geometric parameters (Å, °)

**Table:**

Cu—N5	2.036 (3)	C2Q—C3Q	1.372 (6)
Cu—P2	2.212 (1)	C2Q—H2Q	0.93
Cu—P1	2.238 (1)	C6R—C5R	1.383 (6)
Cu—O4	2.466 (3)	C6R—H6R	0.93
Cu—O61	2.529 (3)	C3Q—C4Q	1.379 (7)
P1—C1P	1.800 (4)	C3Q—H3Q	0.93
P1—C1Q	1.812 (4)	C1T—C6T	1.378 (6)
P1—C1R	1.815 (4)	C5Q—C4Q	1.369 (6)
P3—F3	1.563 (3)	C5Q—H5Q	0.93
P3—F5	1.576 (3)	C6S—C1S	1.378 (6)
P3—F1	1.578 (3)	C6S—H6S	0.93
P3—F2	1.580 (3)	C5R—C4R	1.346 (7)
P3—F6	1.585 (3)	C5R—H5R	0.93
P3—F4	1.590 (3)	C4T—C5T	1.369 (6)
P2—C1U	1.809 (4)	C4T—C3T	1.376 (6)
P2—C1S	1.817 (4)	C4T—H4T	0.93
P2—C1T	1.818 (4)	C2S—C3S	1.362 (6)
O2—C2	1.198 (5)	C2S—C1S	1.387 (6)
O4—C4	1.225 (5)	C2S—H2S	0.93
O61—C61	1.209 (5)	C5T—C6T	1.377 (6)
N3—C4	1.372 (5)	C5T—H5T	0.93
N3—C2	1.392 (6)	C6—C61	1.485 (6)
N3—C3	1.468 (6)	C6T—H6T	0.93
N1—C8A	1.366 (5)	C62—C61	1.485 (6)
N1—C2	1.370 (5)	C62—H62A	0.96
N1—C1	1.481 (6)	C62—H62B	0.96
N8—C8A	1.325 (5)	C62—H62C	0.96
N8—C7	1.336 (6)	C1—H1A	0.96
N5—C6	1.324 (5)	C1—H1B	0.96
N5—C4A	1.334 (5)	C1—H1C	0.96
C8A—C4A	1.384 (6)	C3P—C4P	1.363 (7)
C6U—C1U	1.375 (6)	C3P—C2P	1.380 (6)
C6U—C5U	1.380 (6)	C3P—H3P	0.93
C6U—H6U	0.93	C4Q—H4Q	0.93
C2U—C3U	1.369 (6)	C2P—H2P	0.93
C2U—C1U	1.388 (6)	C4R—C3R	1.373 (6)
C2U—H2U	0.93	C4R—H4R	0.93
C7—C6	1.408 (6)	C5U—C4U	1.367 (7)
C7—C71	1.489 (6)	C5U—H5U	0.93
C1Q—C2Q	1.375 (6)	C4P—C5P	1.361 (6)
C1Q—C6Q	1.380 (6)	C4P—H4P	0.93
C1R—C2R	1.388 (6)	C3T—H3T	0.93
C1R—C6R	1.390 (6)	C3—H3A	0.96
C1P—C2P	1.378 (6)	C3—H3B	0.96
C1P—C6P	1.392 (6)	C3—H3C	0.96
C5S—C4S	1.352 (7)	C3S—C4S	1.387 (7)
C5S—C6S	1.373 (6)	C3S—H3S	0.93
C5S—H5S	0.93	C6P—C5P	1.377 (6)
C2T—C1T	1.380 (6)	C6P—H6P	0.93
C2T—C3T	1.389 (6)	C4U—C3U	1.371 (7)
C2T—H2T	0.93	C4U—H4U	0.93
C4A—C4	1.449 (6)	C4S—H4S	0.93
C71—H71A	0.96	C3U—H3U	0.93
C71—H71B	0.96	C5P—H5P	0.93
C71—H71C	0.96	C3R—C2R	1.377 (6)
C6Q—C5Q	1.377 (6)	C3R—H3R	0.93
C6Q—H6Q	0.93	C2R—H2R	0.93
			
P2—Cu—P1	130.68 (4)	N1—C2—N3	116.7 (4)
P1—Cu—O4	91.13 (9)	C2Q—C3Q—C4Q	120.2 (4)
P1—Cu—O61	107.04 (8)	C2Q—C3Q—H3Q	119.9
N5—Cu—P1	108.2 (1)	C4Q—C3Q—H3Q	119.9
P2—Cu—O4	102.85 (9)	C6T—C1T—C2T	119.0 (4)
P2—Cu—O61	88.81 (8)	C6T—C1T—P2	121.1 (3)
N5—Cu—P2	121.1 (1)	C2T—C1T—P2	119.7 (3)
O4—Cu—O61	143.9 (1)	C4Q—C5Q—C6Q	120.4 (4)
O4—Cu—N5	74.3 (1)	C4Q—C5Q—H5Q	119.8
O61—Cu—N5	70.5 (1)	C6Q—C5Q—H5Q	119.8
C1P—P1—C1Q	103.8 (2)	C5S—C6S—C1S	120.3 (5)
C1P—P1—C1R	107.41 (19)	C5S—C6S—H6S	119.8
C1Q—P1—C1R	101.13 (19)	C1S—C6S—H6S	119.8
C1P—P1—Cu	108.38 (14)	C4R—C5R—C6R	120.1 (4)
C1Q—P1—Cu	117.90 (14)	C4R—C5R—H5R	119.9
C1R—P1—Cu	117.02 (14)	C6R—C5R—H5R	119.9
F3—P3—F5	91.82 (17)	C5T—C4T—C3T	120.7 (4)
F3—P3—F1	91.25 (18)	C5T—C4T—H4T	119.7
F5—P3—F1	90.51 (17)	C3T—C4T—H4T	119.7
F3—P3—F2	90.15 (18)	C3S—C2S—C1S	120.8 (5)
F5—P3—F2	178.02 (19)	C3S—C2S—H2S	119.6
F1—P3—F2	89.66 (18)	C1S—C2S—H2S	119.6
F3—P3—F6	89.31 (18)	C4T—C5T—C6T	119.3 (4)
F5—P3—F6	89.44 (17)	C4T—C5T—H5T	120.3
F1—P3—F6	179.4 (2)	C6T—C5T—H5T	120.3
F2—P3—F6	90.37 (17)	N5—C6—C7	120.4 (4)
F3—P3—F4	179.0 (2)	N5—C6—C61	113.6 (4)
F5—P3—F4	88.81 (16)	C7—C6—C61	126.0 (4)
F1—P3—F4	89.51 (17)	C6S—C1S—C2S	118.6 (4)
F2—P3—F4	89.22 (18)	C6S—C1S—P2	122.5 (3)
F6—P3—F4	89.94 (17)	C2S—C1S—P2	118.9 (3)
C1U—P2—C1S	103.53 (19)	C5T—C6T—C1T	121.3 (4)
C1U—P2—C1T	100.84 (19)	C5T—C6T—H6T	119.4
C1S—P2—C1T	105.4 (2)	C1T—C6T—H6T	119.4
C1U—P2—Cu	116.22 (14)	C61—C62—H62A	109.5
C1S—P2—Cu	114.68 (14)	C61—C62—H62B	109.5
C1T—P2—Cu	114.54 (14)	H62A—C62—H62B	109.5
C4—N3—C2	125.2 (4)	C61—C62—H62C	109.5
C4—N3—C3	119.0 (4)	H62A—C62—H62C	109.5
C2—N3—C3	115.8 (4)	H62B—C62—H62C	109.5
C8A—N1—C2	122.9 (4)	N1—C1—H1A	109.5
C8A—N1—C1	119.3 (4)	N1—C1—H1B	109.5
C2—N1—C1	117.7 (4)	H1A—C1—H1B	109.5
C8A—N8—C7	116.8 (4)	N1—C1—H1C	109.5
C6—N5—C4A	117.9 (4)	H1A—C1—H1C	109.5
C6—N5—Cu	123.6 (3)	H1B—C1—H1C	109.5
C4A—N5—Cu	117.8 (3)	C4P—C3P—C2P	120.2 (4)
N8—C8A—N1	118.8 (4)	C4P—C3P—H3P	119.9
N8—C8A—C4A	122.0 (4)	C2P—C3P—H3P	119.9
N1—C8A—C4A	119.2 (4)	C5Q—C4Q—C3Q	119.4 (4)
C1U—C6U—C5U	120.2 (4)	C5Q—C4Q—H4Q	120.3
C1U—C6U—H6U	119.9	C3Q—C4Q—H4Q	120.3
C5U—C6U—H6U	119.9	C1P—C2P—C3P	120.6 (4)
C3U—C2U—C1U	120.8 (4)	C1P—C2P—H2P	119.7
C3U—C2U—H2U	119.6	C3P—C2P—H2P	119.7
C1U—C2U—H2U	119.6	C5R—C4R—C3R	120.5 (4)
N8—C7—C6	121.4 (4)	C5R—C4R—H4R	119.8
N8—C7—C71	115.6 (4)	C3R—C4R—H4R	119.8
C6—C7—C71	123.0 (4)	C4U—C5U—C6U	120.2 (5)
C2Q—C1Q—C6Q	118.8 (4)	C4U—C5U—H5U	119.9
C2Q—C1Q—P1	119.5 (3)	C6U—C5U—H5U	119.9
C6Q—C1Q—P1	121.6 (3)	C5P—C4P—C3P	120.6 (4)
C6U—C1U—C2U	118.8 (4)	C5P—C4P—H4P	119.7
C6U—C1U—P2	123.8 (3)	C3P—C4P—H4P	119.7
C2U—C1U—P2	116.9 (3)	C4T—C3T—C2T	119.6 (4)
C2R—C1R—C6R	118.2 (4)	C4T—C3T—H3T	120.2
C2R—C1R—P1	125.1 (3)	C2T—C3T—H3T	120.2
C6R—C1R—P1	116.5 (3)	N3—C3—H3A	109.5
C2P—C1P—C6P	118.0 (4)	N3—C3—H3B	109.5
C2P—C1P—P1	122.2 (3)	H3A—C3—H3B	109.5
C6P—C1P—P1	118.9 (3)	N3—C3—H3C	109.5
C4S—C5S—C6S	120.6 (5)	H3A—C3—H3C	109.5
C4S—C5S—H5S	119.7	H3B—C3—H3C	109.5
C6S—C5S—H5S	119.7	C2S—C3S—C4S	119.5 (5)
C1T—C2T—C3T	120.2 (4)	C2S—C3S—H3S	120.2
C1T—C2T—H2T	119.9	C4S—C3S—H3S	120.2
C3T—C2T—H2T	119.9	C5P—C6P—C1P	121.0 (4)
N5—C4A—C8A	121.0 (4)	C5P—C6P—H6P	119.5
N5—C4A—C4	117.8 (4)	C1P—C6P—H6P	119.5
C8A—C4A—C4	121.2 (4)	C5U—C4U—C3U	120.2 (4)
O4—C4—N3	122.6 (4)	C5U—C4U—H4U	119.9
O4—C4—C4A	122.8 (4)	C3U—C4U—H4U	119.9
N3—C4—C4A	114.6 (4)	C5S—C4S—C3S	120.1 (4)
C7—C71—H71A	109.5	C5S—C4S—H4S	120
C7—C71—H71B	109.5	C3S—C4S—H4S	120
H71A—C71—H71B	109.5	C2U—C3U—C4U	119.8 (5)
C7—C71—H71C	109.5	C2U—C3U—H3U	120.1
H71A—C71—H71C	109.5	C4U—C3U—H3U	120.1
H71B—C71—H71C	109.5	C4P—C5P—C6P	119.6 (4)
C5Q—C6Q—C1Q	120.5 (4)	C4P—C5P—H5P	120.2
C5Q—C6Q—H6Q	119.8	C6P—C5P—H5P	120.2
C1Q—C6Q—H6Q	119.8	C4R—C3R—C2R	120.3 (5)
C3Q—C2Q—C1Q	120.8 (4)	C4R—C3R—H3R	119.8
C3Q—C2Q—H2Q	119.6	C2R—C3R—H3R	119.8
C1Q—C2Q—H2Q	119.6	C3R—C2R—C1R	120.2 (4)
C5R—C6R—C1R	120.6 (4)	C3R—C2R—H2R	119.9
C5R—C6R—H6R	119.7	C1R—C2R—H2R	119.9
C1R—C6R—H6R	119.7	O61—C61—C6	118.3 (4)
O2—C2—N1	122.9 (4)	O61—C61—C62	121.5 (4)
O2—C2—N3	120.3 (4)	C6—C61—C62	119.7 (4)
			
N5—Cu—P1—C1P	23.80 (18)	C2R—C1R—C6R—C5R	−0.4 (6)
P2—Cu—P1—C1P	−158.14 (15)	P1—C1R—C6R—C5R	174.7 (3)
N5—Cu—P1—C1Q	−93.63 (19)	C8A—N1—C2—O2	−179.4 (4)
P2—Cu—P1—C1Q	84.43 (17)	C1—N1—C2—O2	−3.1 (6)
N5—Cu—P1—C1R	145.35 (19)	C8A—N1—C2—N3	1.4 (5)
P2—Cu—P1—C1R	−36.59 (17)	C1—N1—C2—N3	177.7 (4)
N5—Cu—P2—C1U	20.5 (2)	C4—N3—C2—O2	−178.0 (4)
P1—Cu—P2—C1U	−157.32 (16)	C3—N3—C2—O2	1.6 (6)
N5—Cu—P2—C1S	141.42 (19)	C4—N3—C2—N1	1.2 (6)
P1—Cu—P2—C1S	−36.43 (17)	C3—N3—C2—N1	−179.2 (3)
N5—Cu—P2—C1T	−96.56 (18)	C1Q—C2Q—C3Q—C4Q	−1.0 (7)
P1—Cu—P2—C1T	85.59 (15)	C3T—C2T—C1T—C6T	−0.3 (6)
P2—Cu—N5—C6	−80.8 (3)	C3T—C2T—C1T—P2	174.4 (3)
P1—Cu—N5—C6	97.5 (3)	C1U—P2—C1T—C6T	22.1 (4)
P2—Cu—N5—C4A	108.6 (3)	C1S—P2—C1T—C6T	−85.3 (4)
P1—Cu—N5—C4A	−73.1 (3)	Cu—P2—C1T—C6T	147.7 (3)
C7—N8—C8A—N1	−171.8 (4)	C1U—P2—C1T—C2T	−152.5 (3)
C7—N8—C8A—C4A	7.7 (6)	C1S—P2—C1T—C2T	100.1 (4)
C2—N1—C8A—N8	178.5 (4)	Cu—P2—C1T—C2T	−26.9 (4)
C1—N1—C8A—N8	2.2 (6)	C1Q—C6Q—C5Q—C4Q	0.0 (7)
C2—N1—C8A—C4A	−1.0 (6)	C4S—C5S—C6S—C1S	−1.2 (7)
C1—N1—C8A—C4A	−177.3 (4)	C1R—C6R—C5R—C4R	1.0 (7)
C8A—N8—C7—C6	−4.2 (6)	C3T—C4T—C5T—C6T	−0.2 (7)
C8A—N8—C7—C71	176.2 (4)	C4A—N5—C6—C7	6.7 (6)
C1P—P1—C1Q—C2Q	−133.2 (4)	Cu—N5—C6—C7	−163.9 (3)
C1R—P1—C1Q—C2Q	115.6 (4)	C4A—N5—C6—C61	−170.6 (4)
Cu—P1—C1Q—C2Q	−13.3 (4)	Cu—N5—C6—C61	18.8 (5)
C1P—P1—C1Q—C6Q	51.2 (4)	N8—C7—C6—N5	−3.1 (6)
C1R—P1—C1Q—C6Q	−60.1 (4)	C71—C7—C6—N5	176.5 (4)
Cu—P1—C1Q—C6Q	171.0 (3)	N8—C7—C6—C61	173.8 (4)
C5U—C6U—C1U—C2U	−1.7 (6)	C71—C7—C6—C61	−6.6 (7)
C5U—C6U—C1U—P2	170.3 (3)	C5S—C6S—C1S—C2S	−0.2 (7)
C3U—C2U—C1U—C6U	3.0 (6)	C5S—C6S—C1S—P2	−178.5 (4)
C3U—C2U—C1U—P2	−169.5 (4)	C3S—C2S—C1S—C6S	2.1 (7)
C1S—P2—C1U—C6U	18.9 (4)	C3S—C2S—C1S—P2	−179.6 (4)
C1T—P2—C1U—C6U	−90.0 (4)	C1U—P2—C1S—C6S	−116.1 (4)
Cu—P2—C1U—C6U	145.5 (3)	C1T—P2—C1S—C6S	−10.7 (4)
C1S—P2—C1U—C2U	−169.0 (3)	Cu—P2—C1S—C6S	116.2 (4)
C1T—P2—C1U—C2U	82.1 (4)	C1U—P2—C1S—C2S	65.6 (4)
Cu—P2—C1U—C2U	−42.4 (4)	C1T—P2—C1S—C2S	171.1 (3)
C1P—P1—C1R—C2R	−23.5 (4)	Cu—P2—C1S—C2S	−62.0 (4)
C1Q—P1—C1R—C2R	84.9 (4)	C4T—C5T—C6T—C1T	−0.6 (7)
Cu—P1—C1R—C2R	−145.6 (3)	C2T—C1T—C6T—C5T	0.8 (6)
C1P—P1—C1R—C6R	161.7 (3)	P2—C1T—C6T—C5T	−173.8 (3)
C1Q—P1—C1R—C6R	−89.8 (4)	C6Q—C5Q—C4Q—C3Q	0.6 (7)
Cu—P1—C1R—C6R	39.6 (4)	C2Q—C3Q—C4Q—C5Q	−0.1 (7)
C1Q—P1—C1P—C2P	−148.2 (4)	C6P—C1P—C2P—C3P	−0.6 (6)
C1R—P1—C1P—C2P	−41.6 (4)	P1—C1P—C2P—C3P	−169.8 (4)
Cu—P1—C1P—C2P	85.7 (4)	C4P—C3P—C2P—C1P	1.1 (7)
C1Q—P1—C1P—C6P	42.7 (4)	C6R—C5R—C4R—C3R	−0.7 (7)
C1R—P1—C1P—C6P	149.3 (3)	C1U—C6U—C5U—C4U	−0.4 (7)
Cu—P1—C1P—C6P	−83.4 (3)	C2P—C3P—C4P—C5P	−0.1 (7)
C6—N5—C4A—C8A	−3.3 (6)	C5T—C4T—C3T—C2T	0.7 (7)
Cu—N5—C4A—C8A	167.9 (3)	C1T—C2T—C3T—C4T	−0.4 (6)
C6—N5—C4A—C4	173.8 (4)	C1S—C2S—C3S—C4S	−2.5 (7)
Cu—N5—C4A—C4	−15.0 (5)	C2P—C1P—C6P—C5P	−0.8 (6)
N8—C8A—C4A—N5	−4.2 (6)	P1—C1P—C6P—C5P	168.8 (3)
N1—C8A—C4A—N5	175.3 (4)	C6U—C5U—C4U—C3U	1.3 (7)
N8—C8A—C4A—C4	178.8 (4)	C6S—C5S—C4S—C3S	0.8 (7)
N1—C8A—C4A—C4	−1.7 (6)	C2S—C3S—C4S—C5S	1.1 (8)
C2—N3—C4—O4	178.5 (4)	C1U—C2U—C3U—C4U	−2.2 (7)
C3—N3—C4—O4	−1.1 (6)	C5U—C4U—C3U—C2U	0.0 (7)
C2—N3—C4—C4A	−3.7 (6)	C3P—C4P—C5P—C6P	−1.3 (7)
C3—N3—C4—C4A	176.7 (3)	C1P—C6P—C5P—C4P	1.7 (7)
N5—C4A—C4—O4	4.6 (6)	C5R—C4R—C3R—C2R	−0.2 (7)
C8A—C4A—C4—O4	−178.3 (4)	C4R—C3R—C2R—C1R	0.7 (7)
N5—C4A—C4—N3	−173.2 (4)	C6R—C1R—C2R—C3R	−0.4 (6)
C8A—C4A—C4—N3	3.9 (5)	P1—C1R—C2R—C3R	−175.1 (4)
C2Q—C1Q—C6Q—C5Q	−1.1 (6)	N5—C6—C61—O61	−31.9 (6)
P1—C1Q—C6Q—C5Q	174.6 (3)	C7—C6—C61—O61	151.0 (4)
C6Q—C1Q—C2Q—C3Q	1.6 (7)	N5—C6—C61—C62	140.4 (4)
P1—C1Q—C2Q—C3Q	−174.2 (3)	C7—C6—C61—C62	−36.8 (6)
